# CCTs as new biomarkers for the prognosis of head and neck squamous cancer

**DOI:** 10.1515/med-2020-0114

**Published:** 2020-07-13

**Authors:** Yanbo Dong, Siyu Lu, Zhenxiao Wang, Liangfa Liu

**Affiliations:** Department of Otolaryngology Head and Neck Surgery, Beijing Friendship Hospital, Capital Medical University, 95th Yong’an Road, Xicheng District, Beijing 100050, China; Department of Emergency, Aviation General Hospital, Beijing 100012, China

**Keywords:** chaperonin-containing TCP-1, TRiC, head and neck squamous cancer, prognosis, Kaplan–Meier plot

## Abstract

The chaperonin-containing T-complex protein 1 (CCT) subunits participate in diverse diseases. However, little is known about their expression and prognostic values in human head and neck squamous cancer (HNSC). This article aims to evaluate the effects of CCT subunits regarding their prognostic values for HNSC. We mined the transcriptional and survival data of CCTs in HNSC patients from online databases. A protein–protein interaction network was constructed and a functional enrichment analysis of target genes was performed. We observed that the mRNA expression levels of CCT1/2/3/4/5/6/7/8 were higher in HNSC tissues than in normal tissues. Survival analysis revealed that the high mRNA transcriptional levels of CCT3/4/5/6/7/8 were associated with a low overall survival. The expression levels of CCT4/7 were correlated with advanced tumor stage. And the overexpression of CCT4 was associated with higher N stage of patients. Validation of CCTs’ differential expression and prognostic values was achieved by the Human Protein Atlas and GEO datasets. Mechanistic exploration of CCT subunits by the functional enrichment analysis suggests that these genes may influence the HNSC prognosis by regulating PI3K-Akt and other pathways. This study implies that CCT3/4/6/7/8 are promising biomarkers for the prognosis of HNSC.

## 1 Introduction

Head and neck squamous cancer (HNSC) is the sixth leading malignancy worldwide, with an annual incidence of more than 6,00,000 cases [1]. Despite considerable advancements in diagnostic and treatment methods, the 5 year overall survival (OS) rate of HNSC remains less than 50% [2]. Hence, prognostic markers and potential drug targets should be identified to enhance the prognosis and individualized treatments.

Molecular chaperones are key molecular complexes in the process of correct folding of proteins to produce energetically stable and functionally competent protein conformations. They are essential in maintaining protein homeostasis and proteome integrity, and in cell growth and survival [3]. They can be classified into two mechanistic classes – chaperones such as HSP70 and HSP90, and chaperonins. The eukaryotic chaperonin family includes the type I chaperonin, HSP60, and the type II hetero-oligomeric chaperonin, TRiC (T-complex protein-1 ring complex, also known as CCT). CCT is a multiprotein complex that functions to assist polypeptides in achieving a functional three-dimensional configuration. CCT is estimated to interact with 5–10% of proteome [4] and is the obligate chaperone for both actin and tubulin [5,6,7].

CCT features a cylindrical architecture composed of two rings stacked opposite one another. Each ring contains eight different subunits, namely CCT1–CCT8 [8,9], which recognize different motifs within substrate proteins. The specific arrangement of these subunits provides the ability to fold certain proteins and let them gain expected functions, leading to protein homeostasis. Disrupted protein homeostasis underlies various diseases and conditions including cancers. Researches have shown that CCTs mediate the folding of a number of proteins implicated in oncogenesis such as prooncogenic proteins signal transducer and activator of transcription 3 (STAT3), p53, cell cycle regulatory proteins cell division cycle protein 20 (CDC20), and tumor suppressor Von Hippel–Lindau (VHL) [10,11,12,13].

It is evident that CCT subunits promote the development of several tumor types, including breast cancer [14,15,16], colorectal cancer [17,18,19], uterine sarcoma [20], lung carcinoma [19], ovarian cancer [21], hepatocellular carcinoma (HCC) [22], and multitudinous tumor cells [23,24]. And HSP70, another member of the chaperone family, proves to be a biomarker of head and neck cancer [25]. However, the involvement of CCT subunits in head and neck cancer remains unknown.

To the best of our knowledge, bioinformatics analysis is yet to be applied to explore the role of CCT subunits in HNSC. RNA and DNA research, an essential component of the biological and biomedical studies, has been revolutionized with the development of microarray technology [26]. On the basis of the analyses of thousands of gene expressions or variations in copy numbers published online, we analyzed in detail the expression and mutations of different CCTs in patients with HNSC to determine the expression patterns, potential functions, and distinct prognostic values.

## 2 Materials and methods

### 2.1 mRNA transcriptional levels of CCT subunits

The mRNA transcriptional levels of CCT subunits in cancers were analyzed by the ONCOMINE database (www.oncomine.org). ONCOMINE is currently the world’s largest oncogene chip database and integrated data mining platform [27]. The mRNA expressions of CCTs in clinical cancer specimens were compared with those in normal controls, using a Student’s *t*-test to generate a *P* value. The cutoff of *P* value and fold change were defined as 0.0001 and 2, respectively. The mRNA transcriptional levels of CCTs in HNSC were also analyzed. CCTs expression was assessed in a HNSC tissue relative to its expression in a normal tissue, and the differences associated with *P* ˂ 0.01 were considered significant. Transcriptional levels of CCTs between head and neck cancer tissues and normal samples were also analyzed by Gene Expression Profiling Interactive Analysis (GEPIA) dataset (http://gepia.cancer-pku.cn/). GEPIA is an interactive web server for analyzing the RNA sequencing expression data from The Cancer Genome Atlas (TCGA) project, using a standard processing pipeline [28]. The unmatched normal and tumor tissues were compared. The raw data were filtered based on the cutoffs |log2FC| > 1 and *P* < 0.01. The log2(TPM + 1) transformed expression data were used for plotting and one-way ANOVA for differential analysis.

### 2.2 Relationship between the mRNA levels of CCTs and clinicopathological parameters

To identify the comparability between the tumor group and the normal group of TCGA HNSC, we downloaded the level-3 RNA sequence (RNA Seq) data (fragments per kilobase of transcript per million mapped reads upper quartile data) of HNSC and the corresponding normal tissue samples from the TCGA database (https://www.cancer.gov/tcga). Clinical characteristics of patients from the tumor group and the normal group were compared. The Fisher exact test and *t*-test were used, respectively, to determine the differences in gender and age between patients in different groups. Furthermore, the impact of age and gender on each CCT mRNA expression was calculated by the LinkedOmics database. The LinkedOmics database (http://www.linkedomics.org/login.php) is a web-based platform for analyzing TCGA datasets [29]. The statistical methods were Pearson correlation test and *t*-test for age and gender, respectively.

The expression of CCTs with tumor stage for HNSC was also analyzed using GEPIA databases. The statistical method for differential gene expression analysis was one-way ANOVA. Relationship between the mRNA expression levels and patients’ N stage was analyzed by the LinkedOmics database. The method for differential gene expression analysis is Jonckheere’s trend test.

### 2.3 Survival analysis

The prognostic value of CCTs mRNA expression was evaluated using the LinkedOmics database. The HNSC patient samples were split into two groups by median expression (high vs low expression) and assessed by Kaplan–Meier survival plots and log-rank test. In cases when two survival curves crossed each other – a clear violation of the assumption of proportional hazard rates for log-rank test [30], a two-stage procedure was used. The two-stage procedure includes the log-rank test as the first stage and a proposed procedure for addressing the crossing hazard rates as the second stage [31]. In brief, we set the significance level of the two-stage procedure at 0.05. If the *P* value in the first stage (*P*1) was less than 0.0253, then the total *P* value = *P*1; otherwise, the *P* value in the second stage (*P*2) was calculated, and the total *P* value = 0.0253 + (1 − 0.0253) × *P*2. This procedure was conducted using the R package ComparisonSurv.

### 2.4 Validation of CCTs’ expression level and prognostic value

IHC images of CCTs protein expression in clinical specimens of patients with HNSC and normal tissues were obtained from the Human Protein Atlas database (https://www.proteinatlas.org/) [32]. The HNSC datasets of GSE30784 and GSE41613 were obtained from the NCBI Gene Expression Omnibus (GEO) (https://www.ncbi.nlm.nih.gov/geo/). GSE30784 consists of 167 HNSC samples, 17 dysplasia samples, and 45 normal samples. This dataset was used to validate the expression level of CCTs between tumor and normal samples. GSE41613 consists of 97 HNSC patients with follow-up information. The R packages affy and annotate were used to process the raw data, make expression matrix, and match the probe to their gene symbol. Patient samples were split into two groups by median expression (high vs low expression) and assessed by the log-rank test and two-stage procedure, as mentioned above. Survival curves and the expression level of CCTs were visualized by GraphPad^®^ PRISM version 8.0 (Graph Pad Software, Inc., La Jolla, CA, USA) with *P* values.

### 2.5 TCGA data and cBioPortal

TCGA had both sequencing and pathological data on 30 different cancers. The head and neck squamous cell carcinoma (TCGA, Firehose Legacy) dataset including data from 530 cases with pathology reports was selected for further analyses of CCTs using cBioPortal (www.cbioportal.org) [33,34]. The genomic profiles included mutations, copy-number variance from GISTIC, mRNA expression *z*-scores (RNA Seq V2 RSEM), and protein expression *z*-scores (reverse-phase protein arrays). Pearson correlation coefficient of co-expression was calculated according to the cBioPortal’s online instruction and visualized by R environment. For the other comparisons of correlations between CCTs mRNA transcriptional levels, the co-expression heatmaps with Pearson correlation coefficients were calculated and visualized by R packages – ggcorrplot and ggthemes.

### 2.6 Pathway commons and STRING

Pathway commons (https://www.pathwaycommons.org) is an integrated resource of publicly available information about biological pathways [35]. The Search Tool for the Retrieval of Interacting Genes (STRING) is an online database used to predict PPIs, which are essential for interpreting the molecular mechanisms of key cellular activities in carcinogenesis. In this study, Pathway Commons was used to identify the 50 most frequently altered neighbor genes of the CCTs family and the STRING database was used to build a PPI network of these genes mentioned above. Cytoscape (version 3.7.1), an open source bioinformatics software platform, was used to visualize molecular interaction networks [36].

### 2.7 Functional and pathway enrichment analysis

The Database for Annotation, Visualization, and Integrated Discovery (DAVID, https://david.ncifcrf.gov/) was used to perform the gene ontology (GO) enrichment analysis. DAVID is an online tool for systematic and integrative annotation and enrichment analysis that reveals the biological meaning related to large gene lists [37]. GO analysis for the cellular component (CC), biological process (BP), and molecular function (MF) categories [38] and Kyoto Encyclopedia of Genes and Genomes (KEGG) pathway enrichment analysis [39] were performed for the selected genes (CCTs and the 50 most frequently altered neighbor genes) using the DAVID. A *P* value < 0.05 was considered statistically significant. The results were visualized using R packages.

## 3 Results

### 3.1 mRNA transcriptional levels of CCTs in patients with HNSC

**Figure 1:**
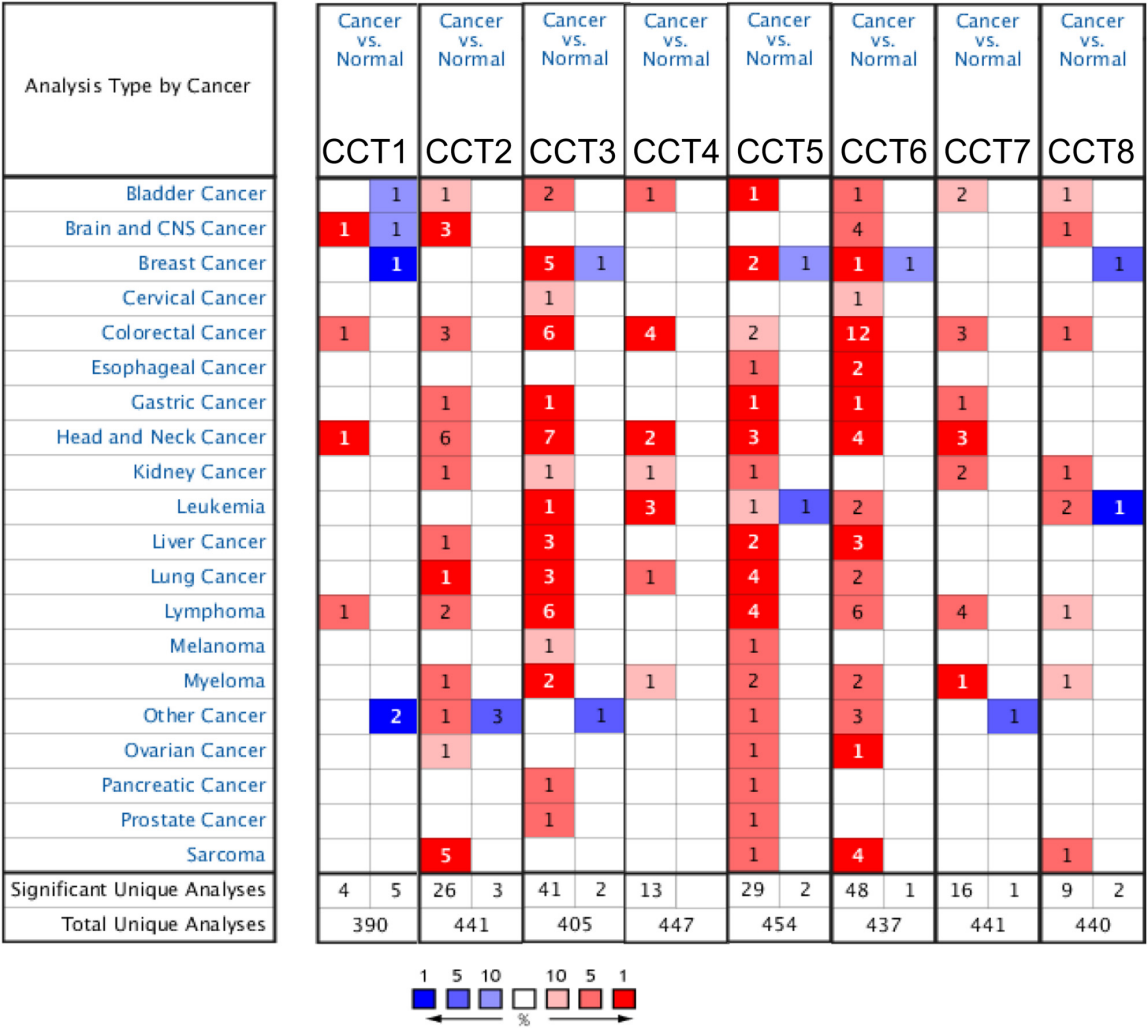
The mRNA transcriptional levels of CCTs in different types of cancers (ONCOMINE). This graphic was generated from ONCOMINE, indicating the numbers of datasets with statistically significant (*P* < 0.0001) mRNA overexpression (red) or downexpression (blue) of CCTs (different types of cancers vs corresponding normal tissue). Cell color was determined by the best gene rank percentile for the analyses within the cell, and the gene rank was analyzed by the percentile of target gene in the top of all genes measured in each research.

Eight CCT subunits have been identified in mammalian cells. We compared the transcriptional levels of CCTs in cancers with those in normal samples by using ONCOMINE databases (Figure 1). Red cell with a number in it indicated the number of datasets with a statistically significant (*P* < 0.0001) mRNA overexpression of CCTs in different types of cancers vs the corresponding normal tissue. There are 13 datasets regarding HNSC in total on ONCOMINE. Statistical details of Figure 1 are presented in Table 1. For instance, the mRNA expression levels of CCT3 were significantly upregulated in patients with HNSC in seven datasets. According to Pyeon’s study [40], CCT3 was overexpressed, compared with those in the normal samples in all of the HNSC types: tonsillar carcinoma with a fold change of 2.534, oral cavity carcinoma with a fold change of 3.115, floor of the mouth carcinoma with a fold change of 3.68, oropharyngeal carcinoma with a fold change of 3.001, and tongue carcinoma with a fold change of 2.529 (Table 1). In Estilo’s study [41] and Talbot’s study [42], CCT3 was also overexpressed in tongue squamous cell carcinoma with fold changes of 2.143 and 2.117, respectively. Similarly, the comparisons of other CCTs mRNA expression levels in HNSC and normal tissues are listed in Table 1. No significant difference was found between the mRNA transcriptional levels of CCT8 in head and neck cancer tissues and normal samples.

**Table 1:** The significant changes in CCTs expression in mRNA transcriptional level between different types of head and neck squamous cancers and normal tissues (ONCOMINE database)

	Types of head and neck squamous cancers vs normal tissue	Fold change	*P* value	*t*-Test	Ref.
CCT1	Oral cavity carcinoma	2.505	7.21 × 10^−6^	5.987	Pyeon [40]
CCT2	Tongue squamous cell carcinoma	2.104	5.29 × 10^−9^	6.749	Estilo[41]
	Tongue squamous cell carcinoma	2.161	1.45 × 10^−9^	7.065	Talbot [42]
	Oropharyngeal carcinoma	3.137	2.43 × 10^−5^	5.405	Pyeon [40]
	Floor of the mouth carcinoma	3.146	9.32 × 10^−6^	5.823	Pyeon [40]
	Tongue carcinoma	2.122	7.05 × 10^−5^	4.437	Pyeon [40]
CCT3	Tonsillar carcinoma	2.534	6.24 × 10^−5^	4.52	Pyeon [40]
	Oral cavity carcinoma	3.115	8.2 × 10^−6^	5.543	Pyeon [40]
	Floor of the mouth carcinoma	3.68	9.87 × 10^−7^	6.287	Pyeon [40]
	Oropharyngeal carcinoma	3.001	6.02 × 10^−5^	4.755	Pyeon [40]
	Tongue carcinoma	2.529	2.44 × 10^−5^	4.795	Pyeon [40]
	Tongue squamous cell carcinoma	2.143	1.94 × 10^−8^	6.605	Estilo [41]
	Tongue squamous cell carcinoma	2.117	1.00 × 10^−7^	6.051	Talbot [42]
CCT4	Floor of the mouth carcinoma	3.035	9.47 × 10^−8^	7.405	Pyeon [40]
	Oropharyngeal carcinoma	2.76	2.89 × 10^−5^	5.516	Pyeon [40]
CCT5	Tongue squamous cell carcinoma	3.009	1.64 × 10^−12^	8.836	Talbot [42]
	Tongue squamous cell carcinoma	2.965	1.38 × 10^−11^	8.354	Estilo [41]
	Floor of the mouth carcinoma	2.142	6.40 × 10^−7^	9.206	Pyeon [40]
CCT6	Nasopharyngeal carcinoma	2.287	5.59 × 10^−6^	6.21	Sengupta [73]
	Floor of the mouth carcinoma	2.845	2.31 × 10^−5^	6.025	Pyeon [40]
	Tongue carcinoma	2.362	8.23 × 10^−6^	5.027	Pyeon [40]
CCT7	Floor of the mouth carcinoma	2.872	8.37 × 10^−8^	7.159	Pyeon [40]
	Oral cavity carcinoma	2.113	2.95 × 10^−5^	4.922	Pyeon [40]
	Oropharyngeal carcinoma	2.273	5.85 × 10^−5^	4.731	Pyeon [40]
CCT8	NA	NA	NA	NA	NA

### 3.2 Relationship between the mRNA levels of CCTs and the clinicopathological parameters of patients with HNSC

**Figure 2:**
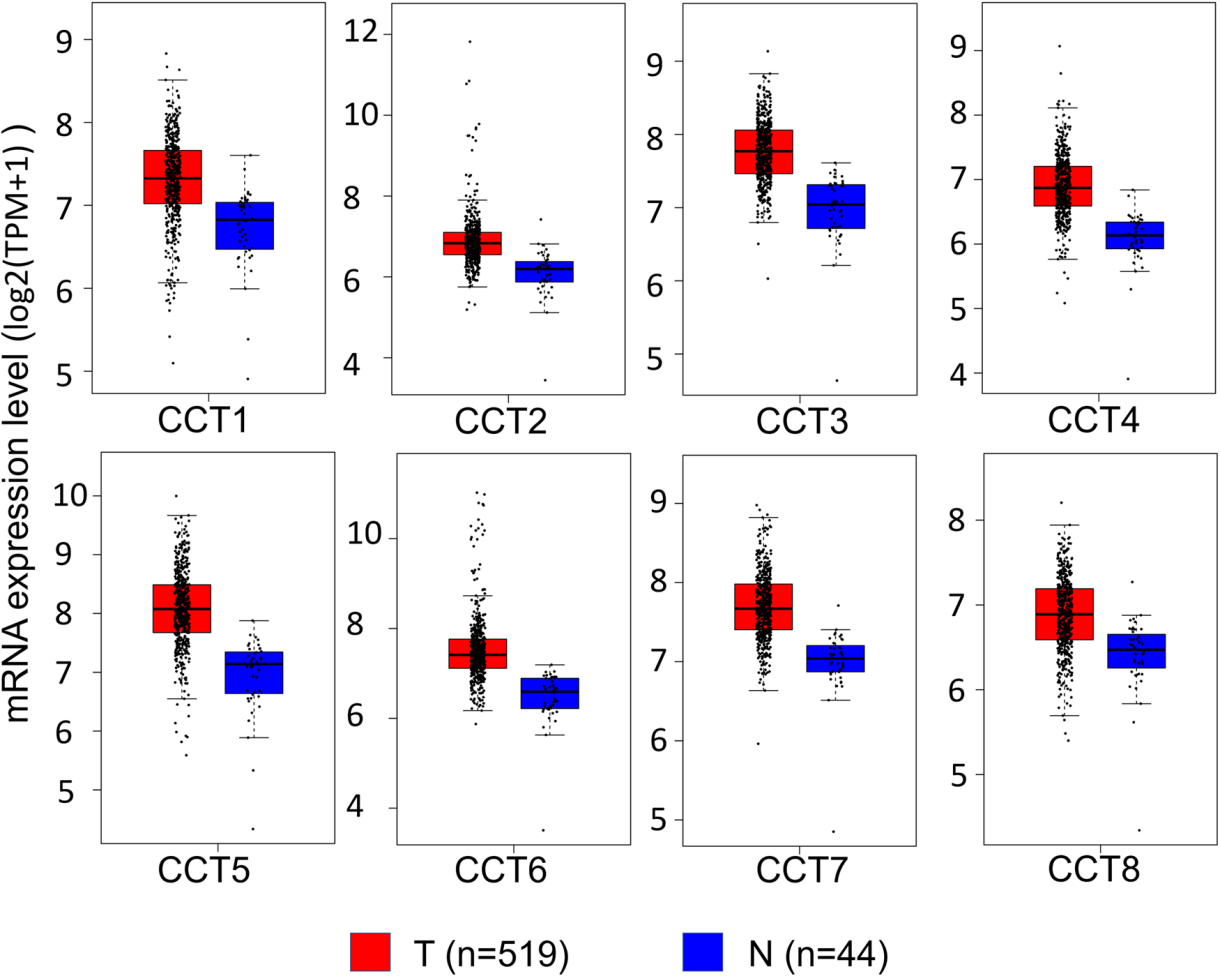
The expression level of CCTs in HNSC (GEPIA). Boxplots showing the relative expression of eight CCTs in HNSC and normal samples. The red and blue boxes represent cancer and normal tissues, respectively.

Using GEPIA dataset (http://gepia.cancer-pku.cn/), we compared the mRNA expression of CCTs between head and neck cancer tissues and normal samples. The results indicated that the expression levels of CCTs were higher in head and neck cancer tissues than in normal tissues (Figure 2). To identify the comparability between the tumor group and the normal group of TCGA HNSC, clinical characteristics of patients from the tumor group and the normal group were compared. There were no differences in gender or age between patients in the two groups (Table S1, ESI). Furthermore, the impact of age and gender on each CCT mRNA expression in TCGA HNSC samples was calculated. The results showed that CCT4 mRNA expression was related to gender statistically, with a higher expression level in male than in female (Table S2).

**Figure 3:**
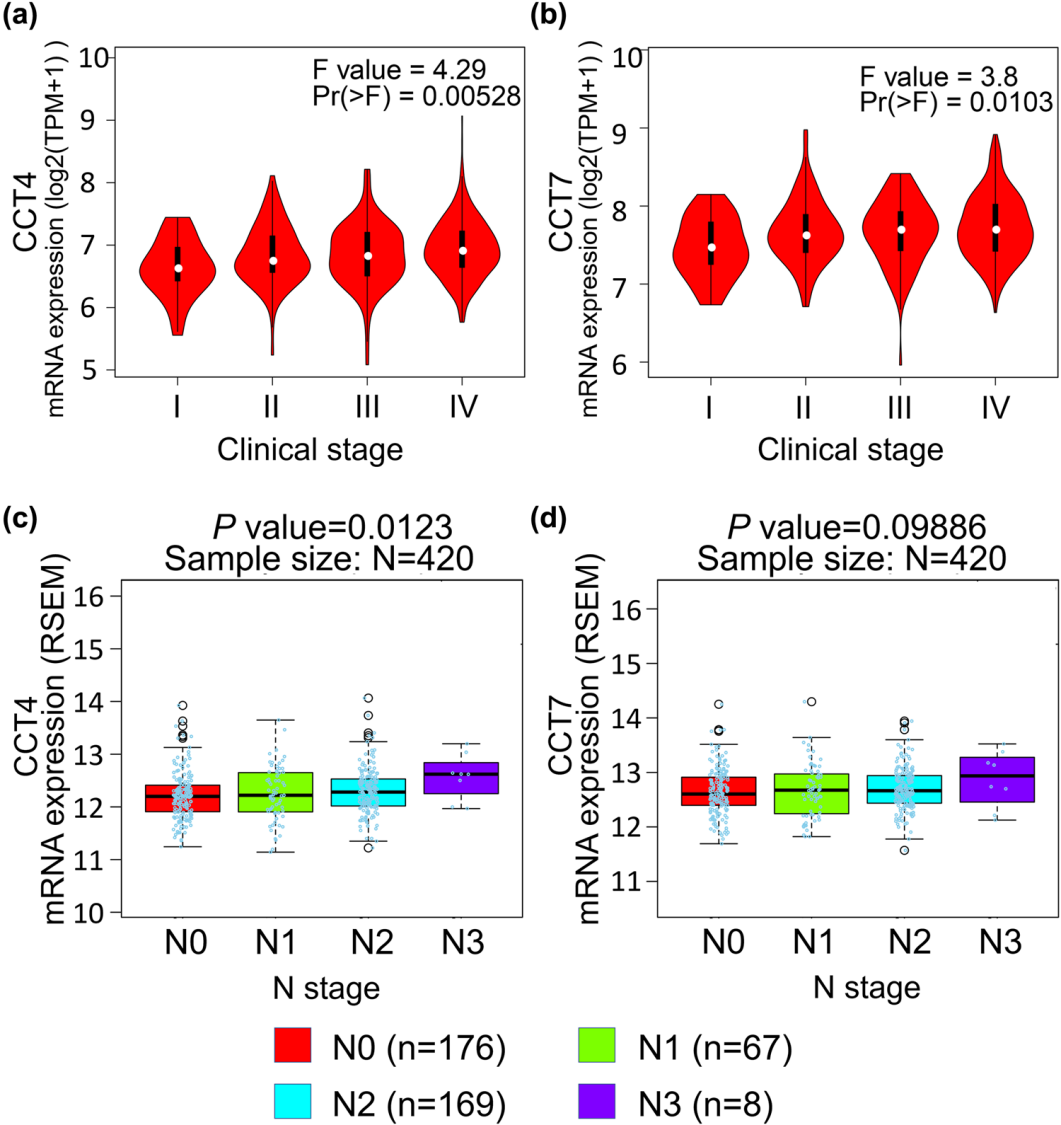
Correlation between the CCT4/7 expression and tumor stage in HNSC patients (GEPIA and LinkedOmics). (a and b) Violin plots showing the relative expression of CCT4/7 in HNSC patients in stage I, II, III, or IV. (c and d) Boxplots indicating the relative expression of CCT4/7 in HNSC patients with N stage of 0, 1, 2, or 3.

The expression of CCTs with tumor stage for HNSC was also analyzed. CCT4 and CCT7 groups significantly varied, whereas the other groups did not significantly differ (Figure 3a and b and Supplement Figure 1). Furthermore, there were differences for CCT4 (*P* = 0.0123) and CCT7 (*P* = 0.09886) in the mRNA expression levels and patients’ N stage, a staging system developed by the American Joint Committee on Cancer to classify patients by the involvement of regional lymph nodes (Figure 3c and d).

### 3.3 Association of the decreased mRNA expression of CCTs with the improved prognosis of patients with HNSC

**Figure 4:**
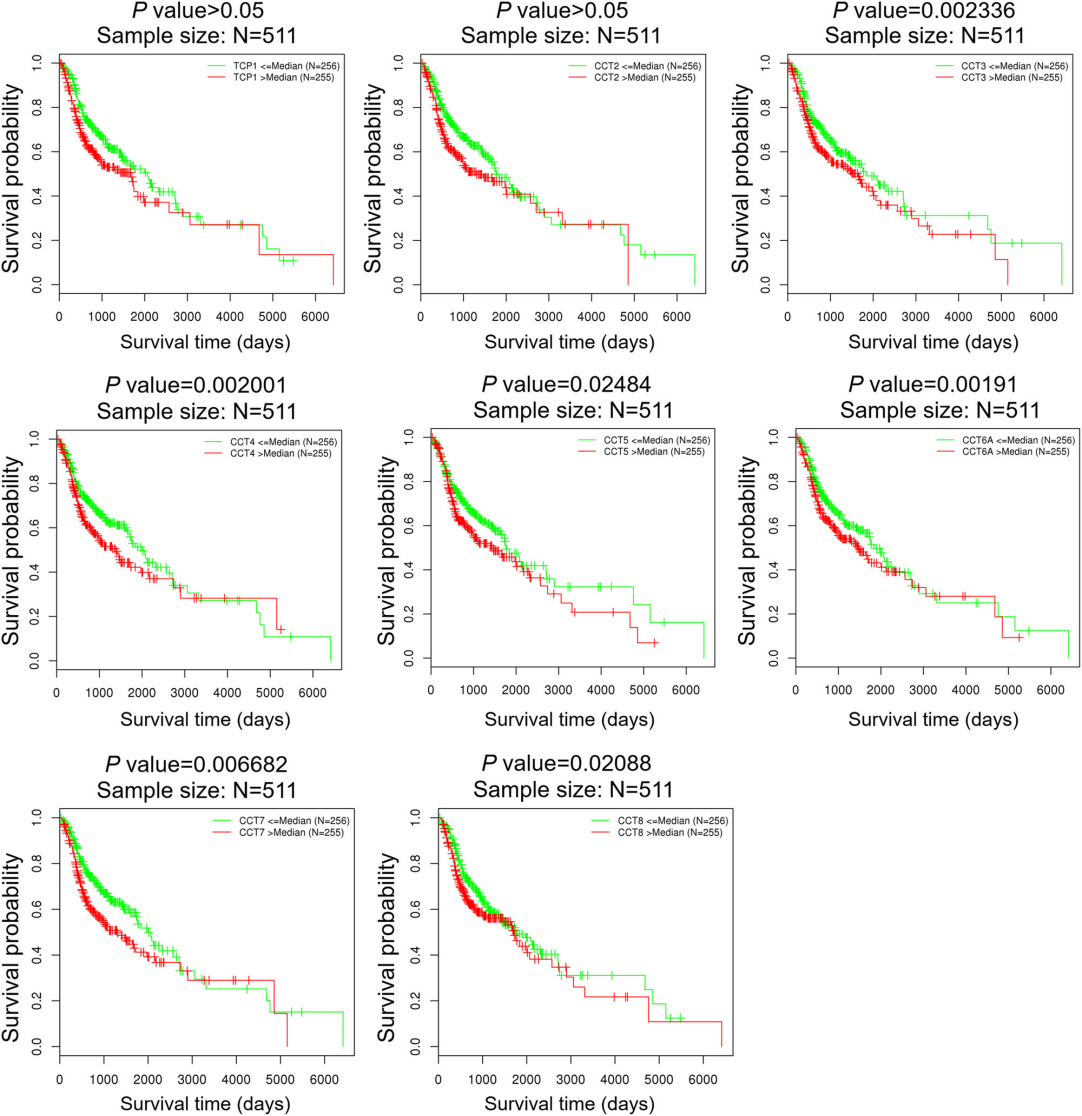
The prognosis value of CCTs in HNSC patients (LinkedOmics). The prognosis value of mRNA expression levels of CCTs in HNSC patients, analyzed by LinkedOmics.

We further explored the critical efficiency of CCTs in the survival of patients with HNSC using the LinkedOmics database. The log-rank test and two-stage procedure analyses for Kaplan–Meier curves from LinkedOmics revealed that the decreased CCT3/4/5/6/7/8 mRNA levels were significantly associated with the OS (Figure 4).

### 3.4 Validation of the expression status and prognosis value of CCTs

**Figure 5:**
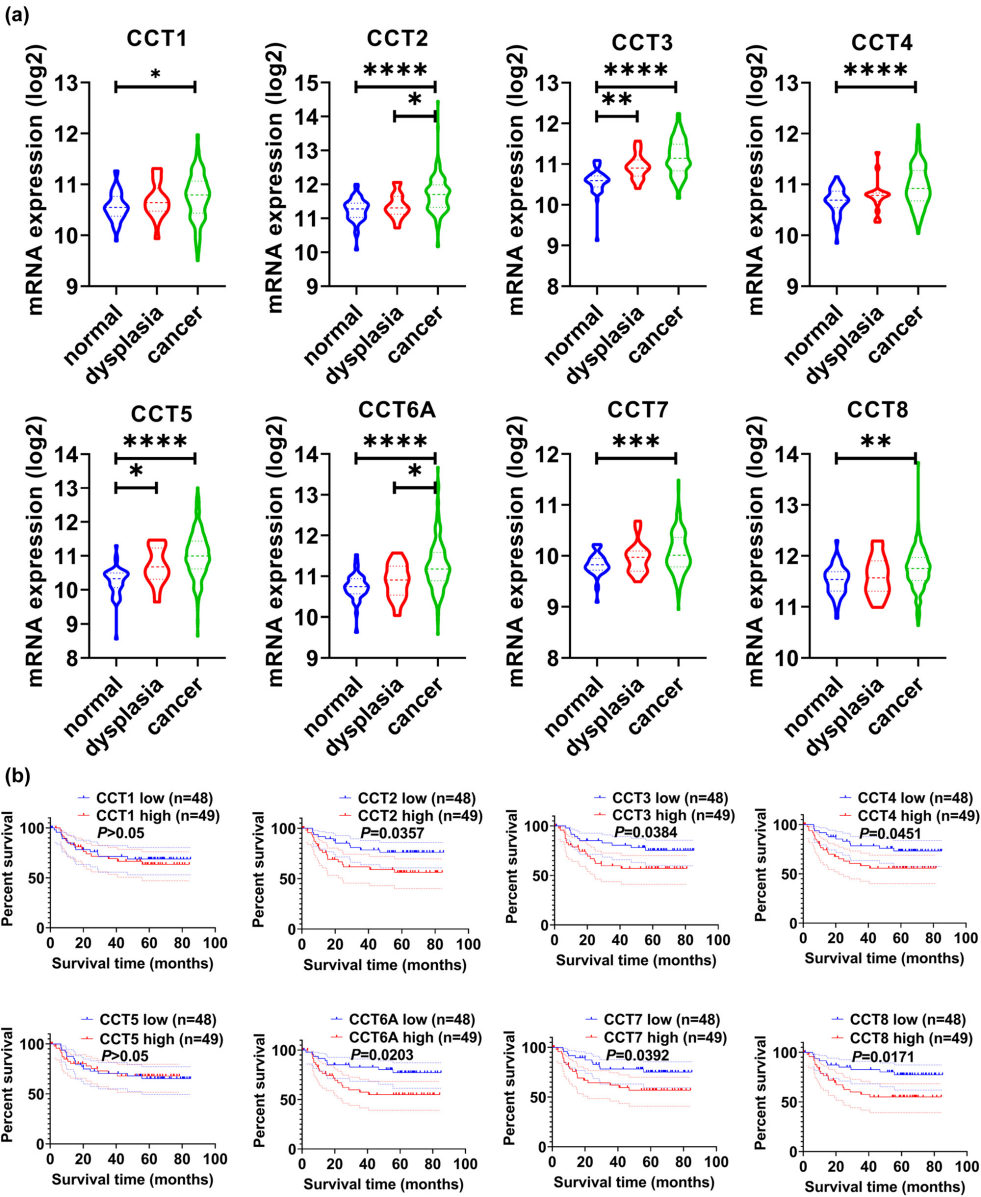
Validation of CCTs’ differential expression and prognostic values using GEO datasets. (a) Validation of CCTs expression level in GSE30784. The expression status of eight CCTs was positively correlated with the disease status. *P < 0.05; **P < 0.01; ***P < 0.001; ****P < 0.0001. (b) Validation of CCTs’ prognostic value in GSE41613. High CCTs expression groups had significantly lower OS than low CCTs expression groups. Shown is the log-rank *P* value.

**Figure 6:**
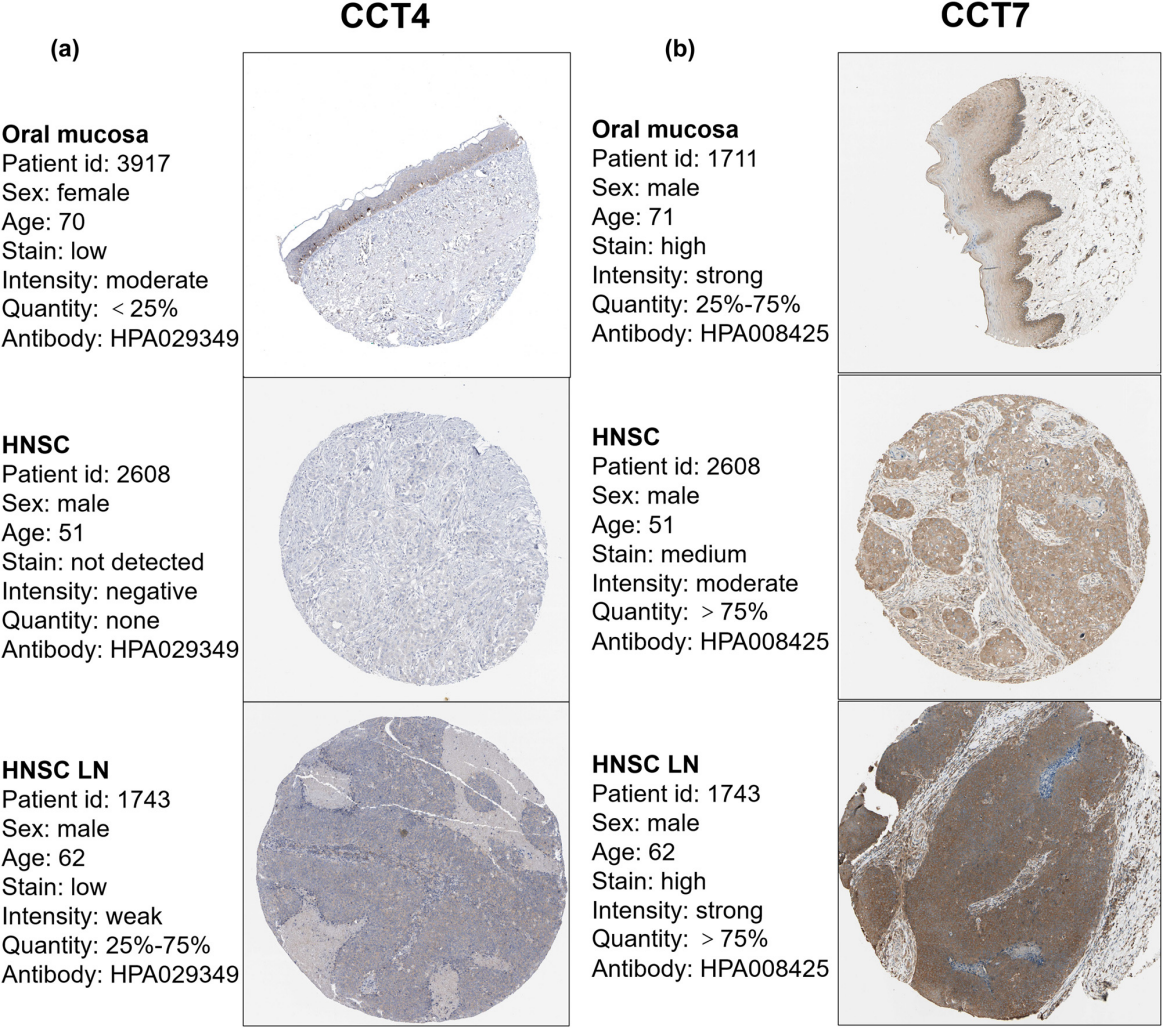
Validation of CCT4/7 in the translational level. IHC staining for CCTs in HNSC and normal tissue from the Human Protein Atlas Portal. (a and b) Validation of CCT4 and CCT7 in HNSC primary site, lymph node metastasis, and normal tissue by The Human Protein Atlas database (IHC).

**Figure 7:**
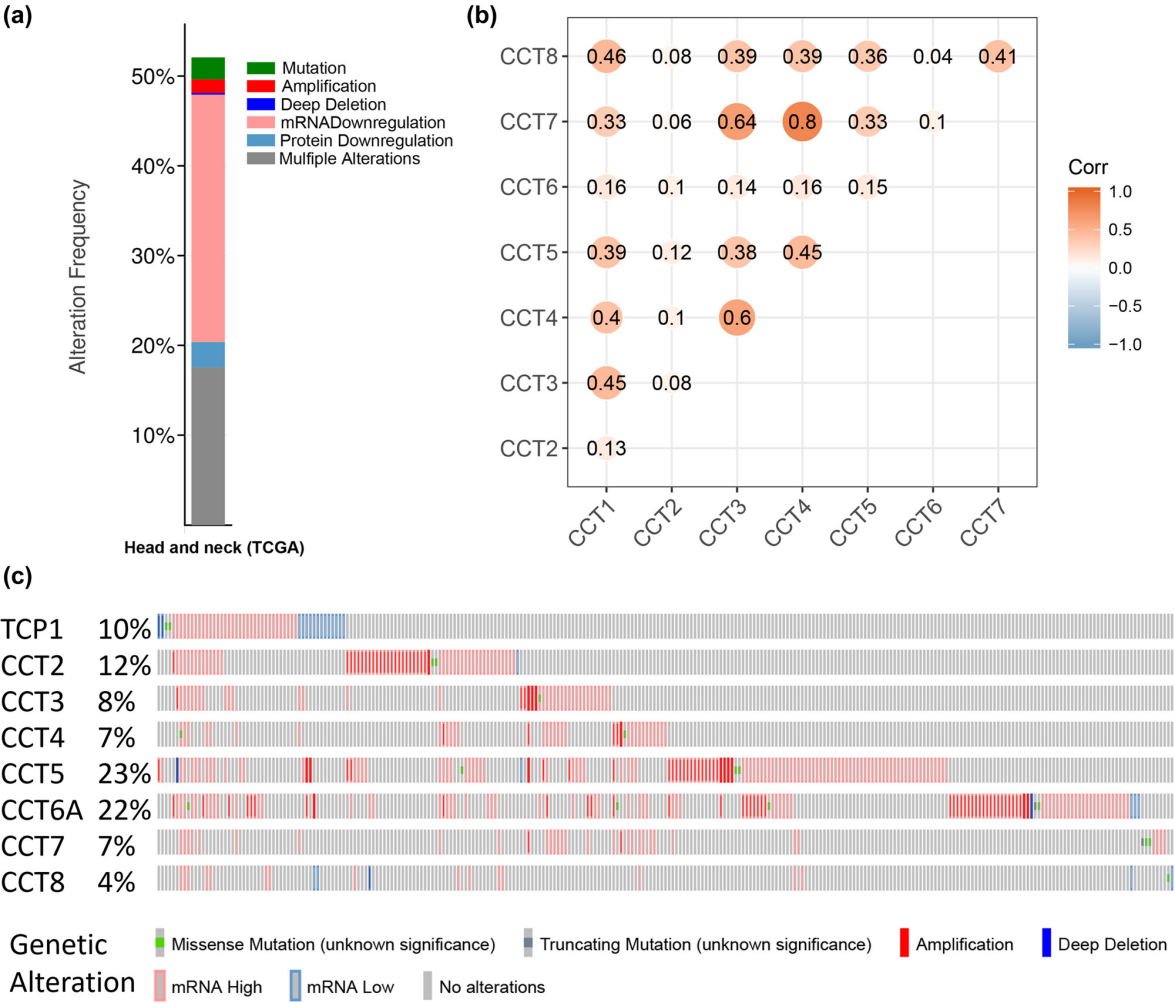
Genetic expression and alteration analysis associated with CCTs in TCGA HNSC (cBioPortal). (a) The total alteration frequency of eight CCT genes in TCGA HNSC is illustrated. (b) Co-expression heatmap of CCT genes in TCGA HNSC, visualized by the R environment. (c) Oncoprint in the cBioPortal represented the proportion and distribution of samples with alterations in CCTs.

We used multiple datasets from the GEO database to validate the expression status and prognosis value of CCTs. Figure 5a demonstrates the expression status of CCT subunits in normal, dysplasia, and OSCC samples of GSE30784. As shown in the figure, the expression status of CCTs was positively correlated with the disease status. Furthermore, GSE41613 was used to validate CCTs’ prognosis value. This dataset contained not only gene transcriptional data but also follow-up information, including 97 patients of HNSC. Patient samples were split into two groups by median expression (high vs low expression) and assessed by the log-rank test and two-stage procedure, as mentioned in the Materials and methods section. Survival analysis was conducted and significant differences were observed between high CCTs and low CCTs groups (Figure 5b). Moreover, immunohistochemistry (IHC) staining obtained from The Human Protein Atlas database also demonstrated the expression status of CCTs family genes and the patient data, as shown in Figure 6 and Supplement Figure 2. We found that CCT2, CCT5, CCT6, and CCT8 proteins were more highly expressed in the HNSC tissues than in the normal tissues (Supplement Figure 2). Notably, the expressions of CCT4 and CCT7 were higher in HNSC lymph node metastasis than in the primary site or normal tissue (Figure 7), which indicated their potential role in tumor metastasis process. These results confirmed that CCTs were closely related to HNSC and were of prognosis value.

### 3.5 Predicted functions and pathways of the changes in CCTs and their frequently altered neighbor genes in patients with HNSC

**Figure 8:**
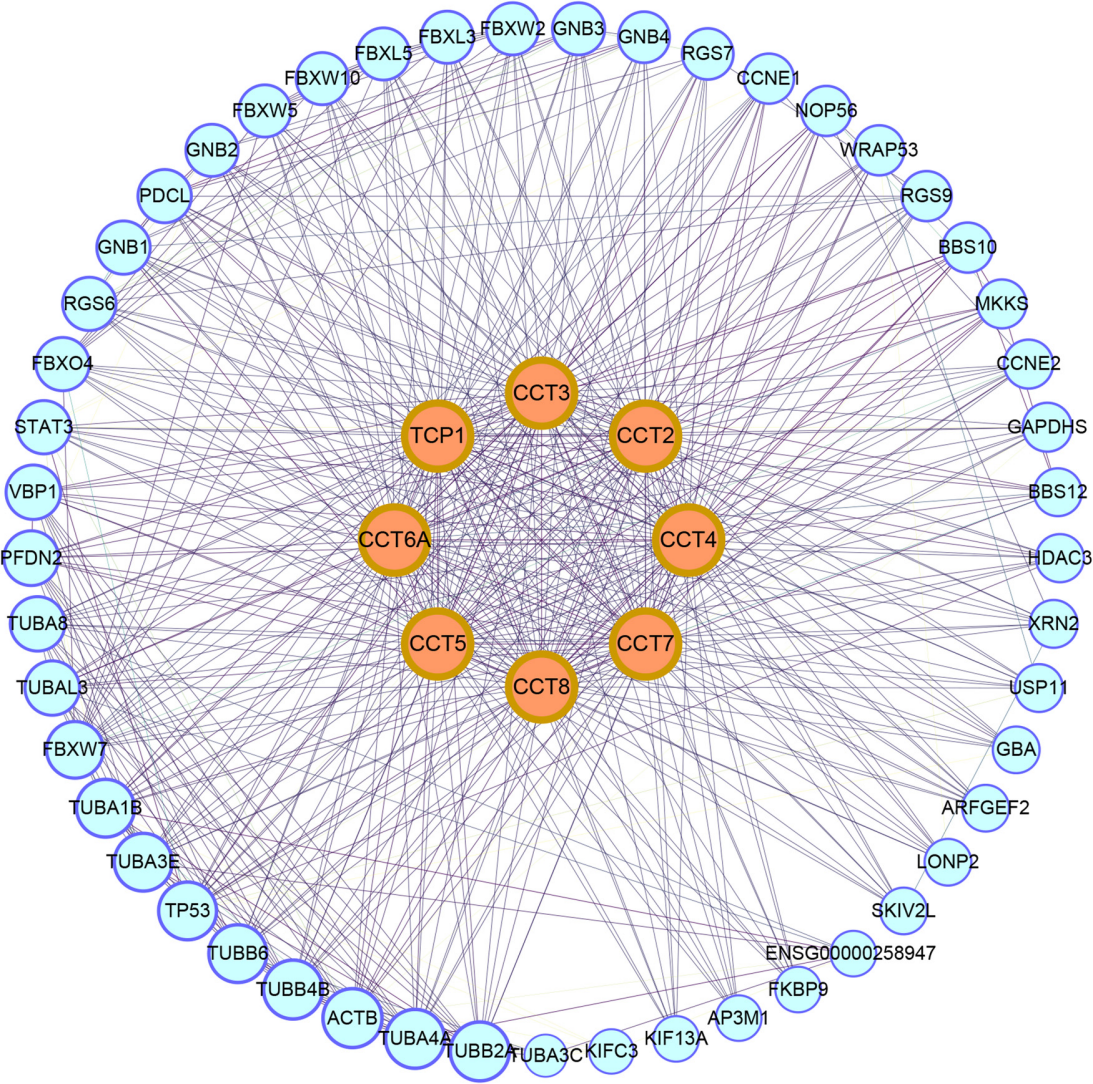
Protein–protein interaction (PPI) network. The PPI network of CCTs and 50 most frequently altered neighbor genes, which includes 58 nodes and 591 edges (PPI enrichment *P* value < 1.0 × 10^−16^), visualized by Cytoscape software.

We analyzed the CCTs alterations by using the cBioPortal online tool for head and neck squamous cell carcinoma (TCGA, Firehose Legacy). CCTs were altered in 276 samples out of 530 patients with HNSC (52%) (Figure 7a). Two or more alterations were detected in almost one third of the samples (93 samples) (Figure 7c). We also calculated the correlations of CCTs with each other by analyzing their mRNA expression (RNA Seq V2 RSEM) via the cBioPortal online tool for head and neck squamous cell carcinoma (TCGA, Firehose Legacy), and Pearson’s correlation was included (Figure 7b). The results indicated significant and positive correlations in the following CCTs: CCT1 with CCT3, CCT4, and CCT8; CCT3 with CCT1, CCT4, and CCT7; CCT4 with CCT1, CCT3, CCT5, and CCT7; CCT7 with CCT3, CCT4, and CCT8; and CCT8 with CCT1 and CCT7 (Figure 7b). More correlation heatmaps are presented in Figure S3, which calculated and compared CCT subunits’ correlations in head and neck normal tissues (Figure S3A), in another dataset GSE41613 (Figure S3B), and at different cancer stages (Figure S3C and D). We then constructed the network for CCTs and the 50 most relevant genes on Pathway Commons (https://www.pathwaycommons.org) [35]. In detail, we input the eight CCT subunits to Pathway Commons and chose HNSC as the cancer type of interest. Seventy-seven relevant genes were calculated and presented by the website ranking by relevance. We chose 50 most relevant genes for further analysis. And a matched functional protein association network of the 50 genes was visualized on The Search Tool for the Retrieval of Interacting Genes (STRING) (Figure 8). The results showed that cancer pathway-related genes, such as TP53, STAT3, CCNE1, and CCNE2, were closely associated with CCTs alterations, just as previously reported by others [43].

**Figure 9:**
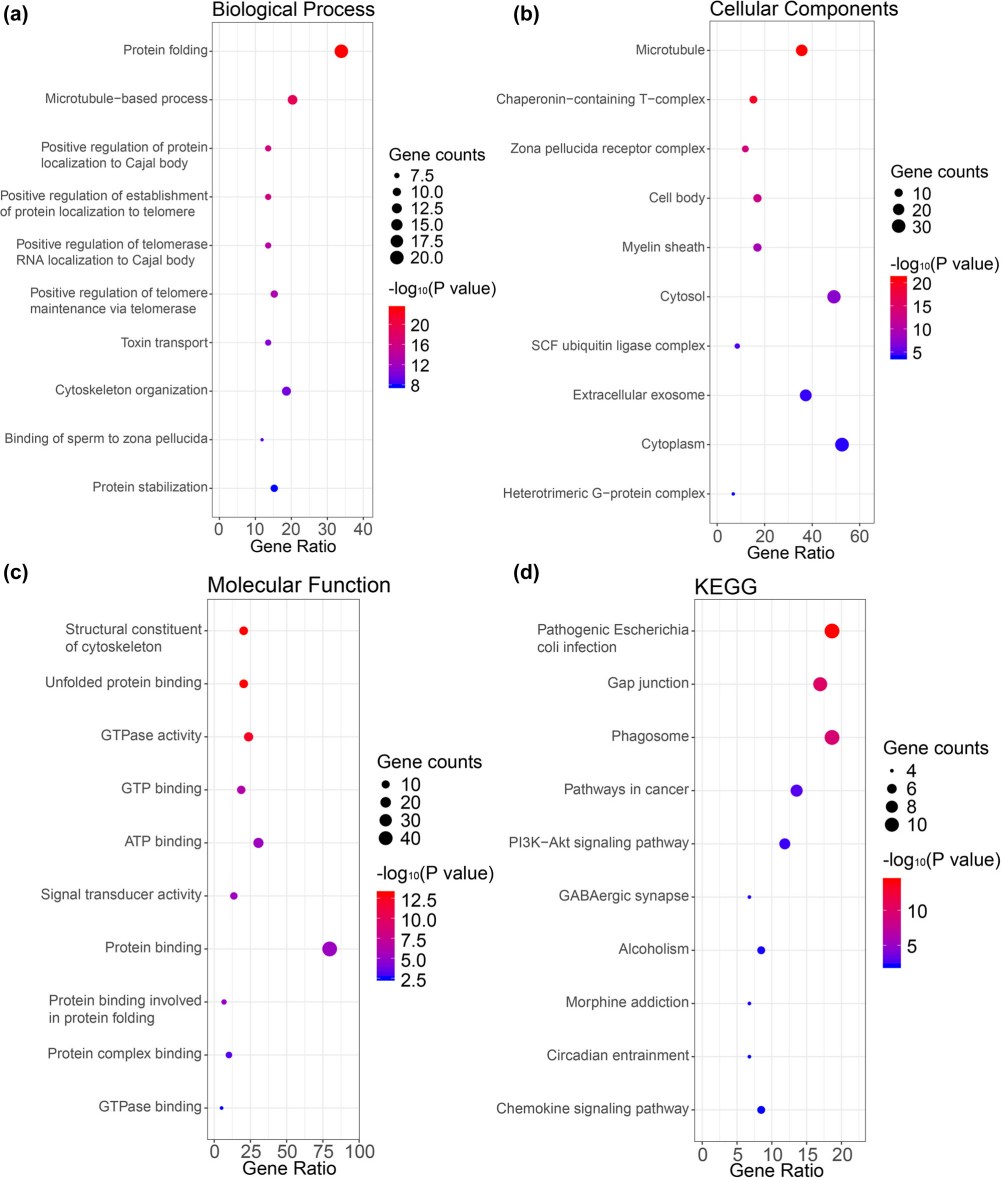
Functional enrichment analysis of CCTs and neighbor genes in HNSC. The bubble diagrams display the enrichment results of CCTs and top 50 genes altered in the CCTs neighborhood in HNSC. (a) Biological processes. (b) Cellular components. (c) Molecular functions. (d) KEGG pathways.

The functions of CCTs and the genes significantly associated with their alterations were predicted by analyzing GO and KEGG in the DAVID. The GO enrichment analysis predicted the functional roles of target genes on the basis of three aspects, including BPs, CCs, and MFs. We found that protein folding, microtubule-based process, and cytoskeleton organization were significantly regulated by the CCTs alterations in HNSC (Figure 9a and Table S3). These genes were largely related to microtubule and extracellular exosome, and were mostly located in cytoplasm or cytosol (Figure 9b and Table S4). Their MF was significantly related to protein binding and ATP binding (Figure 9c and Table S5). The predicted functions and locations were consistent with CCTs’.

KEGG analysis can define the pathways related to the functions of CCTs alterations and the frequently altered neighbor genes. Ten pathways related to the functions of CCTs alterations in HNSC were found through the KEGG analysis (Figure 9d and Table S6). Among these pathways, gap junction, pathways in cancer, phagosome, and PI3K-Akt signaling pathway were closely related to the tumorigenesis and pathogenesis of cancer.

## 4 Discussion

CCTs dysregulation has been reported in many cancers. To our knowledge, the present study is the first to explore the mRNA expression and prognostic values of different CCT genes in head and neck squamous cancer. We hope that our findings will contribute to the available knowledge, improve treatment designs, and enhance the accuracy of prognosis for patients with HNSC.

In the present study, we applied ONCOMINE and TCGA databases to identify the mRNA expression differences of CCTs in HNSC samples and normal tissues. GEPIA and LinkedOmics contributed to comparing the relationship between the mRNA levels of CCTs and the clinicopathological parameters and prognosis. Multiple GEO datasets were used to validate the expression status and prognosis value of CCTs. IHC staining obtained from the HPA database was used to validate the expression difference on a protein level. Functional enrichment analysis of CCTs and most frequently altered genes predicted by Pathway Commons was performed by DAVID.

ONCOMINE consisted of multiple datasets, including DNA-seq data of TCGA, some data from GEO, and many other datasets from literature [44]. It is difficult for us to make comparisons of its comprehensive data. GEPIA and LinkedOmics were both based on the TCGA RNA-seq data [28,29], so the results from these two databases were merely different. Each of these interactive web servers had advantages and disadvantages in terms of application. For example, LinkedOmics could be used to compare detailed clinical information such as age, gender, N stage, etc., while GEPIA could be used to compare the mRNA expression level between the tumor tissue and the normal tissue. These webs simplified the work of downloading and reprocessing the raw data from TCGA and could serve as good assistants for researchers. Distinguishing from the TCGA data, GEO datasets were used for validation. The comparability between the tumor group and the normal group of TCGA HNSC was identified by comparing patients’ gender and age. The impact of age and gender on each CCT mRNA expression in TCGA HNSC samples was also calculated. The results showed that only the CCT4 mRNA expression was related to gender statistically, while others were not related to gender or age. However, the age distribution and gender composition of the patients were similar among different groups. Hence, gender or age has no impact on the CCTs mRNA expression between groups.

In this study, transcriptional levels of CCTs in cancers with those in normal samples indicated that CCTs’ significant overexpression was observed in various types of cancers, including liver cancer, lung cancer, breast cancer, bladder cancer, and so on. This was consistent with the result of literature review, suggesting that CCTs overexpression was not a tissue-specific phenomenon [15,18,45]. According to GEPIA, the expression level of CCTs in HNSC was all higher than normal tissues, but none of them showed significant differences. The expression levels of CCT4 and CCT7 demonstrated to be significantly associated with the tumor stage of HNSC, and only CCT4 was related to patients’ N stage. Interestingly, most of the CCTs were significantly associated with patients’ OS, as confirmed by GSE41613.

Validation of expression status and prognosis value of CCTs was done using datasets from the GEO database and IHC pictures from the HPA. As shown in Figure 5a, there were significant differences in CCTs expression level among normal, dysplasia, and cancer tissue. The result reflected that the expression status of CCTs was positively correlated with the disease status. On a translational level, the IHC showed that CCT4 and CCT7 were less expressed in HNSC samples than in oral mucosa, but their expressions in lymph node metastasis samples were significantly higher than that of a normal tissue, which further indicated their potential role in cancer migration and metastasis. Furthermore, survival analysis between high and low CCTs groups from the GEO database definitely showed their prognostic values in HNSC.

We cannot ignore the fact that the IHC staining for some CCTs was not higher in HNSC tissues than in normal tissues, despite their mRNA transcription being higher in HNSC tissues. The IHC data were obtained from The Human Protein Atlas database. Although the mRNA expression data of CCTs were from large sample sizes, the IHC data of each CCT were from one or two patients’ tumor tissue. The heterogeneity of biological samples in studying RNA and protein expression levels might be one of the causes. In addition, the translation process from mRNA to proteins might be hampered by mRNA modifications in cancer [46]. The phenomenon of inconsistency of RNA and protein expression levels did exist [47], and further experiments were needed to unveil the possible reasons. Genetic expression and alteration analysis associated with CCTs in TCGA HNSC showed that more than half of the patients possessed at least one CCT subunit alteration. Correlations between CCTs mRNA transcriptional levels in head and neck normal tissues revealed a strong correlation among the eight CCT subunits, which was consistent with their biological function of a multiprotein complex [8]. While the correlation heatmaps in HNSC, from TCGA or GSE41613, differed from those in normal tissues, some CCT subunits barely correlated with each other, suggesting an abnormal function of CCTs in tumor. Such correlations were similar at different cancer clinical stages. Pathway analysis showed that pathways related to the functions of CCTs and neighbor genes included pathways in cancer, PI3K-Akt signaling pathway, gap junction, and phagosome, other than expected pathways such as protein folding and microtubule-based process.

PI3K-Akt signaling pathway was one of the most important pathways in HNSC, which was a critical regulatory axis for cell growth, survival, motility, and metabolism in both normal physiology and cancer. TCGA and other studies revealed that the majority of HNSCCs possess alterations in the PI3K/Akt/mTOR pathway [48,49]. In addition, the members of the PI3K/Akt/mTOR axis interacted with and contributed to the regulation of several other signaling molecules in HNSC, including tumor suppressor, TP53, NF-κB, and MAPK/ERK. Since CCTs were predicted to interact with the PI3K-Akt signaling pathway by a functional enrichment analysis, we presumed that the potential mechanism underlying CCTs’ differential expression and prognostic values in HNSC was a cross talk with the PI3K-Akt pathway. Further experiments were needed to better confirm the findings of this study.

The molecular chaperone network (including chaperons and chaperonins) played a central role in maintaining protein homeostasis and proteome integrity [50,51]. The main substrates for CCT seemed to be involved in the folding of cytoskeletal proteins, such as actins and tubulins [5,6,7], and other intracellular proteins, such as protein phosphatase PP2A regulatory subunit B [52], histone deacetylase [53], and cyclin E1 [54]. In addition, CCT was involved in the regulation of cell cycle progression and cytoskeletal organization [55]. The transcriptions of CCT subunits were apparently increased from the G1/S phase transition to the early phase in the process of cell cycle in murine and human cell lines [13,56]. Reduction of the CCT or CCT subunit could lead to a growth arrest, with a great change in cell morphology and motility [57]. Scientists also deemed that CCT’s function in assisting the folding of actin and tubulin could enhance cell migration related to cancer metastasis [58,59]. Moreover, CCT may be overexpressed in cancer cells [24]. Inevitably, members of the molecular chaperone pathway have been implicated in the development of cancers [60,61,62]. Researches have shown that prooncogenic proteins, such as STAT3, p53, CDC20, and tumor suppressor VHL [10,11,12,13], were mediated by CCTs, which partly explained their role in oncogenesis.

The CCTs are essential for cell survival, correct folding and for the function of diverse proteins. Each CCT subunit is the product of an individual gene [63]. However, at present, it is still unclear whether or not the eight subunits form a single particle and cover all functions, and whether they exist as individual subunits or smaller complexes. For example, CCT4 and CCT5 homo-oligomers have been found to form 8-fold double rings without the other subunits. And the CCT4 or CCT5 oligomer rings exhibited activities of ATP hydrolysis and protein folding, comparable to the TRiC ring [64]. CCT4 associates with the plasma membrane and alters the cell shape [65], whereas CCT5 regulates actin expression via the serum response factor pathway [66]. As increasing information on the individual roles played by CCTs components is being accumulated, the possibility of subunit-specific roles for these proteins in cell growth and tumorigenesis also needs to be considered [67].

CCT subunits are also irregulated in different types of cancers. For instance, CCT1 and CCT2 are upregulated in patients with HCC and colonic cancer and correlate with tumor proliferation and poor prognosis [56]. CCT3 is widely studied in different cancers. CCT3 is overexpressed in gastric cancer, and CCT3 knockdown can also suppress the proliferation and induce cell apoptosis in gastric cancer [68] and papillary thyroid carcinoma (PTC) [45]. CCT4 participates in protein CCT6A as a potential prognostic biomarker in glioblastoma [69]. CCT7 effectively regulates VHL proteostasis, which is responsible for sporadic renal cell carcinoma [70]. CCT8 can promote the migration and invasion of esophageal squamous carcinoma by regulating actin and tubulin [71]. And CCT8 has been reported to be upregulated in colon cancer and HCC [56,72]. These studies concluded, as we do, that CCT subunit expression could be a marker of cancer.

We acknowledge that there were some limitations and shortcomings in this study. First, head and neck squamous cancer consists of various types of cancers derived from different anatomic sites, such as pharynx, oral cavity, tongue, larynx, upper esophagus, etc. They have the same histological type of squamous cell carcinoma, but also have different prognoses and responses to treatment due to anatomic differences. This study was mainly focused on the available data of ONCOMINE and TCGA, whose HNSCs were mostly comprised of cancer from the oral cavity and tongue. Hence, a data bias of the cancer type was difficult to be avoided. Second, this study was mainly conducted by the data mining of an online public database. The results were analyzed by the methodology but not validated fully by experiments. Third, more datasets should be involved to obtain a solid result. The datasets for validation, GSE30784 and GSE41613, were from the same departments and using the same array in analysis. Although they had different sets of data and different roles for validation, datasets with demographic diversity were of better reliability.

Here, we systemically analyzed the expression and prognostic values of CCTs in HNSC and provided a better understanding of the heterogeneity and complexity of the molecular biological properties of HNSC. Our results implied that CCT3/4/6/7/8 were promising prognostic biomarkers for the improvement of HNSC survival and prognostic accuracy.

## Abbreviations

CCT: Chaperonin-containing T-complex protein 1
TRiC: T-complex protein-1 ring complex
HNSC: Head and neck squamous cancer
DAVID: Database for annotation, visualization, and integrated discovery
PPI: Protein–protein interaction
GEPIA: Gene expression profiling interactive analysis
STRING: Search tool for the retrieval of interacting genes
OS: Overall survival
STAT3: Signal transducer and activator of transcription 3
CDC20: Cell cycle regulatory proteins cell division cycle protein 20
VHL: Von Hippel–Lindau
HCC: Hepatocellular carcinoma
TCGA: The cancer genome atlas
LN: Lymph node
IHC: Immunohistochemistry
HPA: Human protein atlas
GO: gene ontology
CC: Cellular component
BP: Biological process
MF: Molecular function
KEGG: Kyoto encyclopedia of genes and genomes
HSP: Heat shock protein
PI3K: Phosphoinositide 3-kinase
Akt: Protein kinase B
mTOR: Mammalian target of rapamycin
NF-κB: Nuclear factor-kappa B
MAPK: Mitogen-activated protein kinase
ERK: Extracellular signal-regulated kinase
